# Creative Thinking and Dyscalculia: Conjectures About a Still Unexplored Link

**DOI:** 10.3389/fpsyg.2021.671771

**Published:** 2021-05-28

**Authors:** Sara Magenes, Alessandro Antonietti, Alice Cancer

**Affiliations:** ^1^Department of Psychology, Università Cattolica del Sacro Cuore, Milan, Italy; ^2^Fraternità e Amicizia Società Cooperativa Sociale ONLUS, Milan, Italy

**Keywords:** creativity, dyscalculia, neurodevelopmental disorders, divergent thinking, inhibition, attention, alternate uses

## Introduction

Research on creativity showed enhanced creative thinking skills in individuals with neurodevelopmental disorders, such as dyslexia (Cancer et al., 2016; Manzoli et al., 2016; Cancer and Antonietti, 2019), autism spectrum disorder (Liu et al., 2011; Kasirer and Mashal, 2014, 2016), and attention-deficit/hyperactivity disorder (ADHD) (White and Shah, 2006, 2011, 2016). In this field the relationships between creativity and dyscalculia are somewhat unexplored. Developmental Dyscalculia (DD) is a specific learning disability affecting the acquisition of numerical-arithmetical abilities in children with normal intelligence and age-appropriate school education (WHO, 2010). It affects 3–6% of the population (Shalev et al., 2001).

A literature search of PsychINFO, Scopus, and Pubmed databases–using the following keywords: “Creativity and dyscalculia,” “Divergent thinking and Dyscalculia;” “Creativity and Math Disorders;” “Divergent thinking and Math disorders”–resulted in only one article which met the criteria (Saeidei and Pirkhaefi, 2020). Saeidei and Pirkhaefi (2020) investigated the role of creativity and memory in students with DD. More precisely, performances of this group of students were compared to those of students without any learning disorder, using the Torrance Test of Creative Thinking (TTCT) and the Kim Karad visual memory test. Results showed significantly lower memory and creative performances of students with DD (i.e., lower originality and elaboration scores). In contrast with such results, in a study by Magenes et al.^1^, who studied a sample of 180 high school students (aged 14–17 years), mathematical skills were found to predict negatively fluency in producing novel ideas, an ability which is often associated to creativity (Guilford, 1967). In accordance with the compensatory hypothesis (Chakravarty, 2009), these findings can suggest that students who fail to acquire a standard way to solve operations or problems tend to look for alternative learning approaches, thus enhancing their creative potential.

However, the role of other components of creativity, beside the fluent and divergent production of ideas, have to be taken into account. Considering creativity as a multifaceted construct involving manifold mechanisms (Simonton, 2000), an important role is played by executive functions, defined as higher-order cognitive processes that enable the control of cognitive, behavioral, and emotional activity, including the subprocesses of inhibition, working memory, shifting (Miyake et al., 2000), and cognitive flexibility (Arán Filippetti and Krumm, 2020). Weaknesses in those processes in individuals with DD could potentially hinder their creative potential.

The present opinion article aims at exploring the hypotheses of a positive relation between creativity and DD. Neurobiological and cognitive correspondences between DD and creative profiles point to shared characteristics which could support an enhanced creative potential in people with mathematical disorders. Theoretical conjectures will be discussed to suggest future empirical investigations.

## Is There a Possible Neurobiological Link Between Creativity and DD?

Creativity is often defined as the ability to produce new ideas in order to achieve uncommon outcomes (Mumford, 2003). As mentioned before, some studies highlighted the role of executive functions in the creative process (i.e., cognitive flexibility and inhibition) (Benedek et al., 2012; Zabelina et al., 2012). According to the neuropsychological model of developing creative mind in children with DD, Pournesaei et al. (2020) reported an association between executive functions and creativity (assessed by TTCT), which in turn resulted to be correlated to memory, visuo-spatial, and perceptual skills.

By moving to the investigation of neurobiological bases of creativity (Benedek et al., 2019), the involvement of executive functions is stressed by studies supporting the key role covered by the pre-frontal cortex (PFC: Colombo et al., 2015), that is devoted to multimodal information, control of voluntary behaviors, and inhibition of inadequate responses (Volle et al., 2013; De Souza et al., 2014; Oldrati et al., 2016). In particular, when a damage in the frontal lobe occurs, creative thinking tends to decrease, as revealed both by a qualitative analysis of patients' expressive productions and by quantitative standardized measures (i.e., TTCT) (Acosta, 2014). In addition, creative people have higher activation of the parietal regions (i.e., angular gyrus-AG), ventral medial pre-frontal cortex (VMPFC), premotor cortex, inferior frontal gyrus (IFG), lateral occipital gyrus, and the precuneus during visual creative tasks (Huang et al., 2012; Aziz-Zadeh et al., 2013; Saggar et al., 2015).

Neuroscientific evidence highlights some neurofunctional correspondences between creative thinking and DD. Neuroimaging findings about DD are not consistent, with some studies showing the hyper-activation of the parietal cortices (AG, left bilateral intraparietal sulcus-IPS) and frontal regions (middle frontal gyrus, IFG), known to be important both for ordinal number processing and for the incorporation of the mental number line (Iuculano et al., 2015; Rosenberg-Lee et al., 2015), in students with DD. Kaufmann et al. (2009) investigated functional activation patterns in response to non-symbolic number processing in children with and without DD aged 9.6 years. Higher activations in children with DD in parietal cortex (IPS, supramarginal gyrus, and AG) were found. A similar investigation was later performed by Rosenberg-Lee et al. (2015) on children with and without DD (mean age: 8.34 years). Children with DD showed hyper-activation during addition and subtraction problems in pre-frontal and parietal cortices. In the same year, Iuculano et al. (2015) investigated brain activity patterns in children with and without DD (7–9 years) in arithmetic problem-solving tasks. Results showed higher activity in frontal and parietal cortices, such as IFG, AG, VMPFC, anterior, and posterior cingulate cortex.

In conclusion, some neural structures involved in DD coincide with those involved in the creative process: AG, IFG, and VMPFC. Specifically, AG, lying in the posterior region of parietal lobe, is part of the default mode network (DMN), which is linked to the generation of ideas through the involvement of mental simulation, which plays a pivotal role in creative thinking (Beaty et al., 2018). More precisely, a higher activation in AG has been shown when participants were asked to find many and uncommon ways to use familiar objects task (alternative uses task-AUT) (Fink et al., 2010; Beaty et al., 2018). Also the IFG, a pre-frontal region associated with the inhibitory control, plays a crucial role. By employing a Go-NoGo task training (Tamm et al., 2002; Hartmann et al., 2015), where participants were asked to press a lever with the index finger as soon as they would see a “Go” stimulus and inhibit their response when presented a “No-Go” stimulus, an association between inhibition and divergent thinking (assessed through AUT), mediated by bilateral tDCS stimulation with the cathode over the right IFG and the anode over the left IFG, emerged (Khalil et al., 2020). Finally, VMPFC, a part of the DMN, is important for creativity as well. Results confirmed a significant positive correlation in Creative Achievement Questionnaire scores (CAQ; Carson et al., 2005) since VMPFC is involved in evaluating creative products and goal-directed new solutions (Ellamil et al., 2012; Aziz-Zadeh et al., 2013). On the basis of the mentioned neurofunctional overlaps, the hypothesis of increased creative abilities in DD students could be supported.

## Are There any Cognitive Similarities Between Creativity and Models of DD?

Traditional neuropsychological models of math learning include regulating processes to recognize numbers, execute operations, and apply calculation procedures. In the triple-code model, Dehaene and Cohen (1995) assumed that numbers are mentally manipulated in Arabic, analogical magnitude, and verbal code. Specifically, the Arabic code is involved in number's reading and writing (i.e., written calculation and judgment of parity). The analogical magnitude code is responsible for the number's representation in a non-verbal way (i.e., subitizing and number sense). Finally, the verbal code is involved in numbers' representation in the lexical, phonological, and syntactic way. The linguistic mechanism is involved in the retrieval of arithmetic facts through the activation of the verbal memory (i.e., multiplications table and operations). So, the verbal short-term memory impairment in students with DD (Swanson, 2011; Peng and Fuchs, 2016; Shen et al., 2018) could enhance the use of unconventional ways of thinking so to generate new strategies to process arithmetic computations and to overcome the use of strict memorization procedures.

In McCloskey's et al. (1985) model, the calculation system is separated both from the comprehension of numbers (input) and the production of numbers (output). Specifically, the calculation system, which aims at performing arithmetic calculations, involves names and functions of operational symbols, execution of the four operations, and arithmetic facts. Temple (1997), following McCloskey's et al. (1985) model, described children with computational difficulties in sequential procedures and reported the case of a student with DD who did not present any kind of difficulty in number sense, but impairment only in calculation procedures. It can be conjectured that students with DD prefer a global information processing, not sequential as required by math calculations. Hence, a holistic vision could be associated with a creative way of thinking (Kampylis and Valtanen, 2010).

As for attentional processes, a deficient inhibitory control in DD could avoid that irrelevant potential stimuli are suppressed and, as a consequence, attention cannot be well-focused on the relevant stimulus (Ashkenazi et al., 2009; Hannula et al., 2010; Swanson, 2011). Defocused attention is an aspect of creativity as well. Specifically, this ability assumes a role in enabling deemed and irrelevant concepts, providing unusual solutions to a problem (Peterson and Carson, 2000; Peterson et al., 2002; Carson et al., 2005).

To sum up, the possible link between creativity and DD could be supported in the light of the following aspects. First, the verbal code impairment in DD could foster the use of unconventional and original strategies to solve math computations. Second, in students with DD the preference for global processing of information could match with a creative, holistic way of thinking. Third, impairments in attentional focus could imply the impossibility of suppressing irrelevant information, in line with the defocused attention of creative people. Based on these assumptions, a possible link between creativity and DD is expected.

## Discussion

This article aimed at analyzing theoretical evidence, based on neuroscientific literature, to take into account the hypothesis of increased creative abilities in students with DD. The association between creativity and mathematical difficulties has been underexplored in the literature. However, a possible link between creativity and DD could be supported by a neurofunctional overlap of neural structures involved in both DD and creative thinking (Ellamil et al., 2012; Aziz-Zadeh et al., 2013; Beaty et al., 2018; Becker et al., 2020). Moreover, cognitive similarities were found between creative thinking and neuropsychological models of DD. More precisely, the cognitive peculiarities of students with DD can stimulate creative thinking, such as: the use of unconventional strategies and procedures to solve arithmetic computations, the preference for global information processing, and an attentional dysfunction that facilitates the processing of (apparently) irrelevant information.

Despite the neuroscientific correspondences discussed in the present article, to date no scientific evidence confirmed an enhanced creative potential in people with DD. To test this hypothesis, the association between creativity and DD should be empirically investigated using standardized measures of creative thinking (e.g., CAQ; TTCT; Carson et al., 2005) and involving a sample of individuals with DD, but without any other comorbidities, so to be compared with matched peers not affected by any neurodevelopmental disorder. Specific ways of processing information by persons with DD–such as global vs. analytical processing, focused vs. defocused attention–are worth investigating as well, so to provide a detailed picture of the mechanisms underlying the creativity-DD connection.

If the hypothesis of enhanced creativity in students with DD would be confirmed, such potential could be recognized and exploited during school activities (Antonietti et al., 2020). We suggest that the recognition and stimulation of creative skills by teachers could improve overall well-being of student with DD and reduce the potential risk of low self-esteem and self-efficacy (Magenes et al., in press). Creativity training have been devised also to promote resilience and support students with neurodevelopmental disorders in school (Cancer et al., 2016, 2020; Cancer and Antonietti, 2017)^1^. Fostering the creative potential of these students could lead them to find alternative strategies to overcome the difficulties associated to the neurodevelopmental disorder and thus to achieve better school outcomes and higher satisfaction in life.

## Author Contributions

AA, SM, and AC conceived the ideas presented. SM and AC wrote the article. AA critically revised the manuscript. All authors contributed to the article and approved the submitted version.

## Conflict of Interest

The authors declare that the research was conducted in the absence of any commercial or financial relationships that could be construed as a potential conflict of interest.

## Footnotes

^1^Magenes, S., Cancer, A., Curti, S., Pradella, C., and Antonietti, A. (under review). Creativity, learning skills, and self-efficacy in vocational school students. *Think Skills Creat*.
